# Supplementary material for: A closed translocation channel in the substrate-free AAA+ ClpXP protease diminishes rogue degradation
Source: Nat Commun. 2023 Nov 10;14:7281. doi: 10.1038/s41467-023-43145-x (PMC10638403; doi:10.1038/s41467-023-43145-x)
Supplement: Supplementary file 1 — Supplementary Information [file 41467_2023_43145_MOESM1_ESM.pdf]

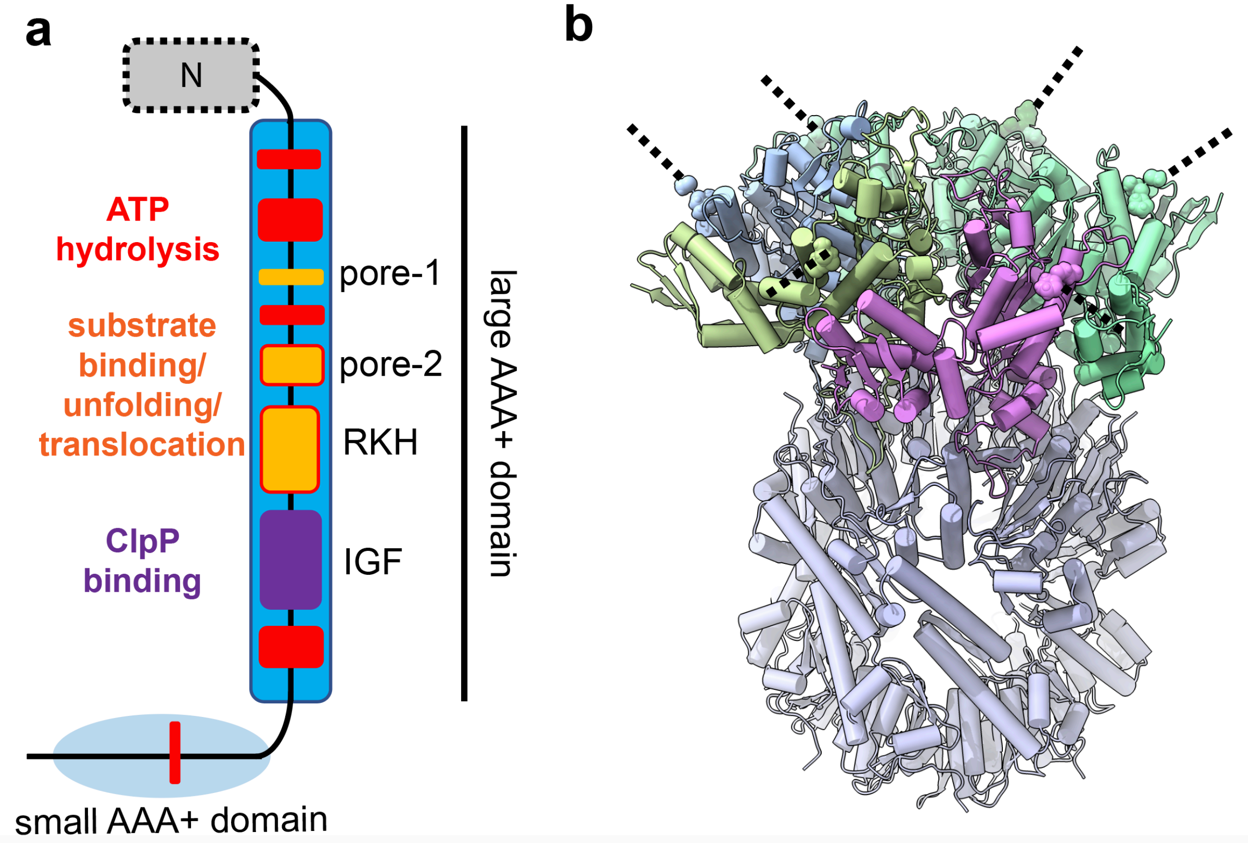

Supplementary Figure 1. ClpX domain architecture. (**a**) Domain structure of ClpX, highlighting the N-domain, large and small AAA+ domains, and regions that are important for ATP binding and hydrolysis (red), protein-substrate binding (orange), and ClpP binding (purple)[3]. (**b**) Positions where flexible linkers to the N-terminal domains of ClpX extend from the large AAA+ domains of the hexamer are indicated by dashed lines.

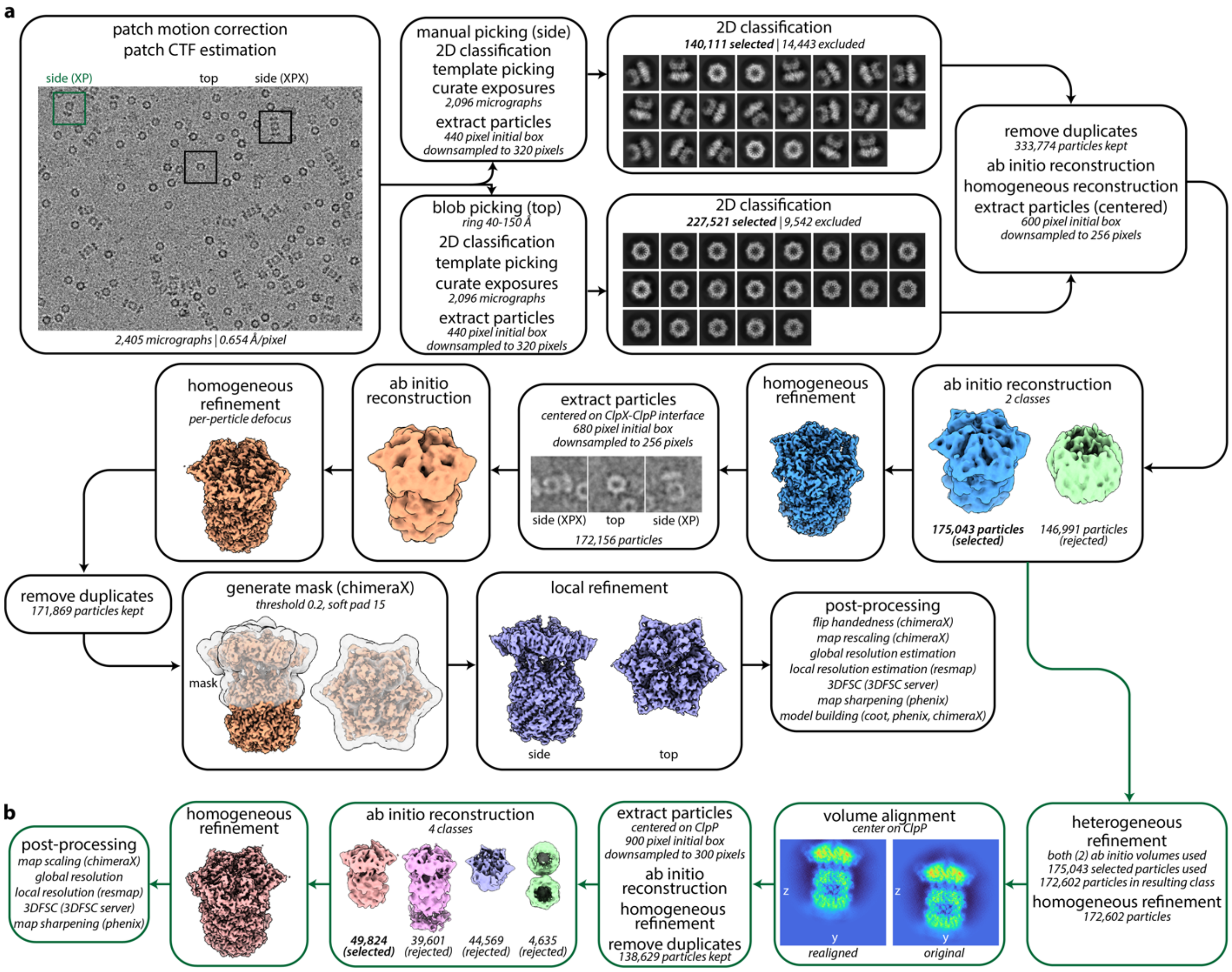

Supplementary Figure 2. CryoSPARC processing workflow for single-chain ClpX$^{\Delta N}$/ClpP particles. (**a**) Single-chain ClpX$^{\Delta N}$/ClpP structures derived from mixtures of singly and doubly capped particles. (**b**) Single-chain ClpX$^{\Delta N}$/ClpP derived from singly capped particles. Note that singly capped particles are an extracted subset of the full particle stack. Job names, job details, and non-default parameters (italicized) are noted in each box.

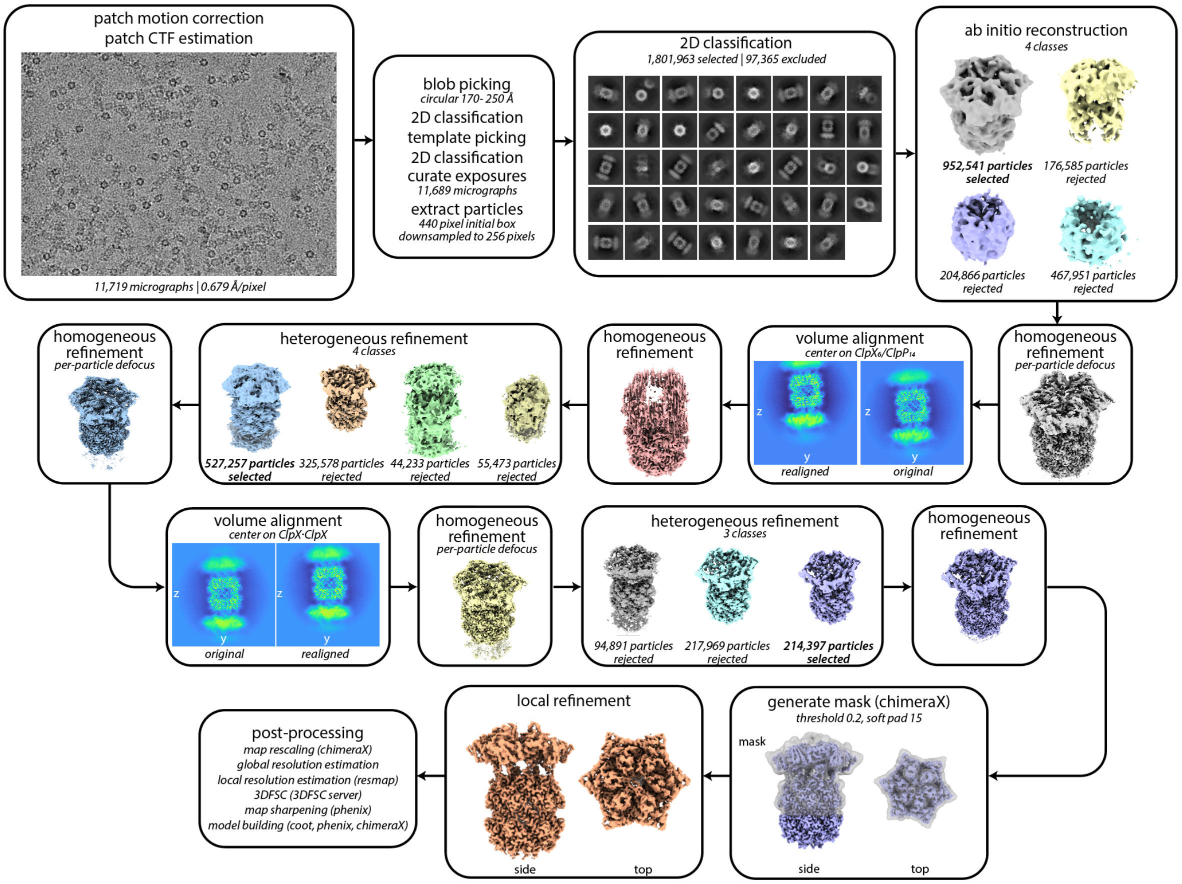

Supplementary Figure 3. CryoSPARC workflow for full-length ClpX/ClpP particle processing. Job names, job details, and non-default parameters (italicized) are noted in each box.

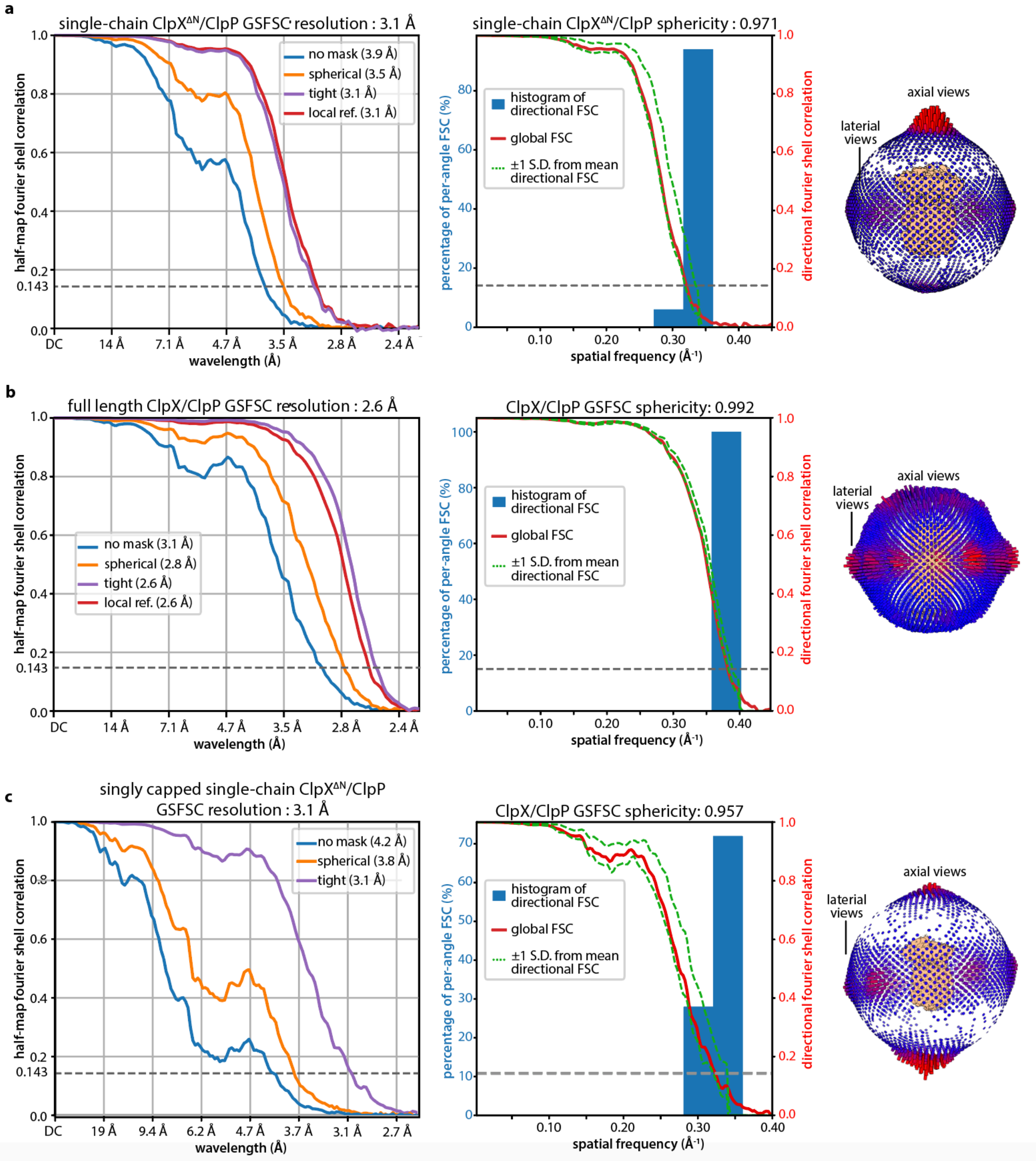

Supplementary Figure 4. Estimates of global resolution, directional resolution, and projection-angle distribution. (**a**) Single-chain ClpX$^{\Delta N}$/ClpP. (**b**) Full-length ClpX/ClpP. (**c**) Single-chain ClpX$^{\Delta N}$/ClpP from singly capped particles.

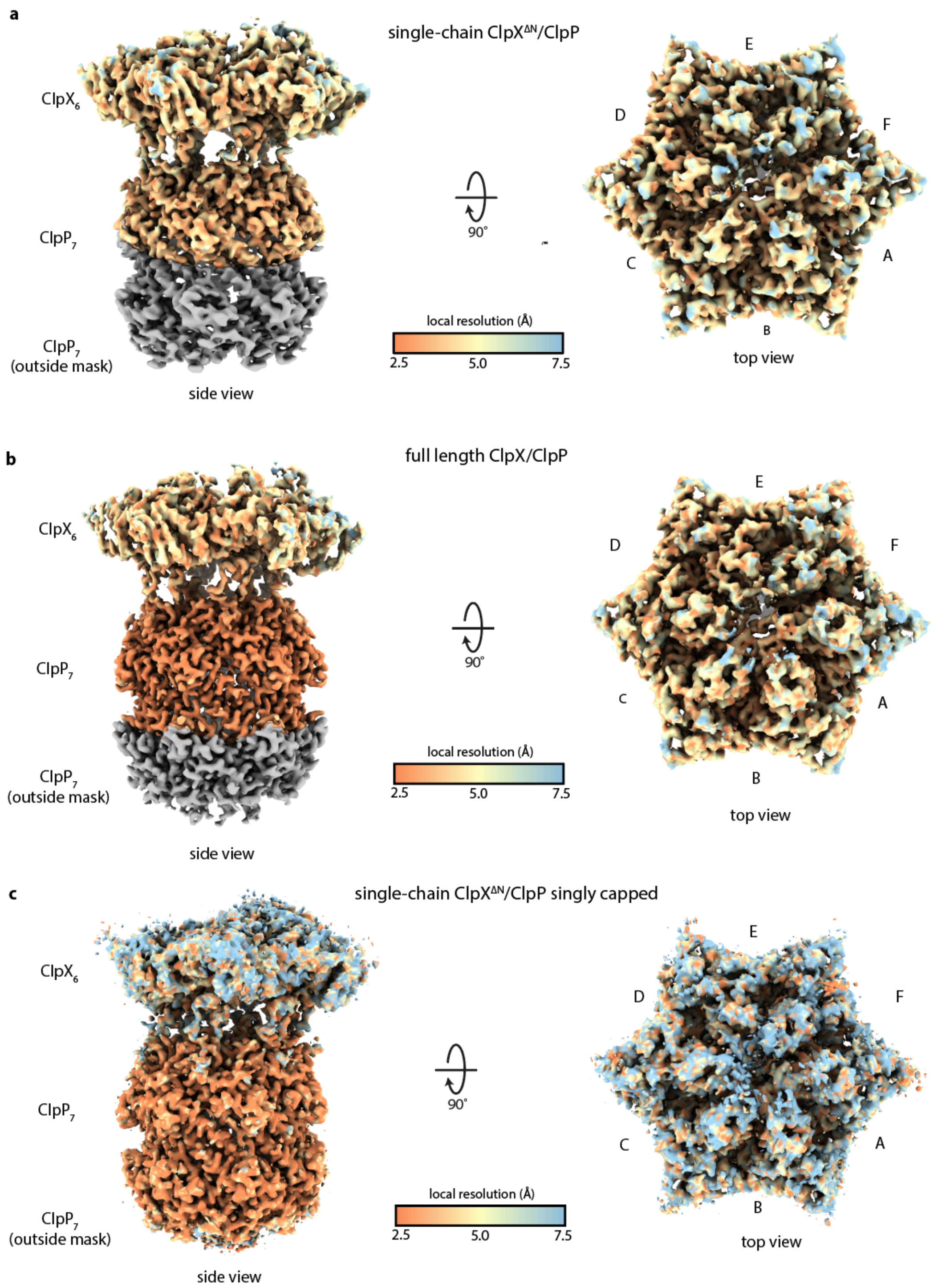

Supplementary Figure 5. Local resolution assessment. Maps colored by local resolution as estimated by cryoSPARC implementation of monoRes[45]. Regions outside of the mask used for local refinement are colored gray. (**a**) Side and top views of single-chain ClpX$^{\Delta N}$/ClpP. (**b**) Side and top views of full-length ClpX/ClpP. (**c**) Side and top views of single-chain ClpX$^{\Delta N}$/ClpP from singly capped particles.

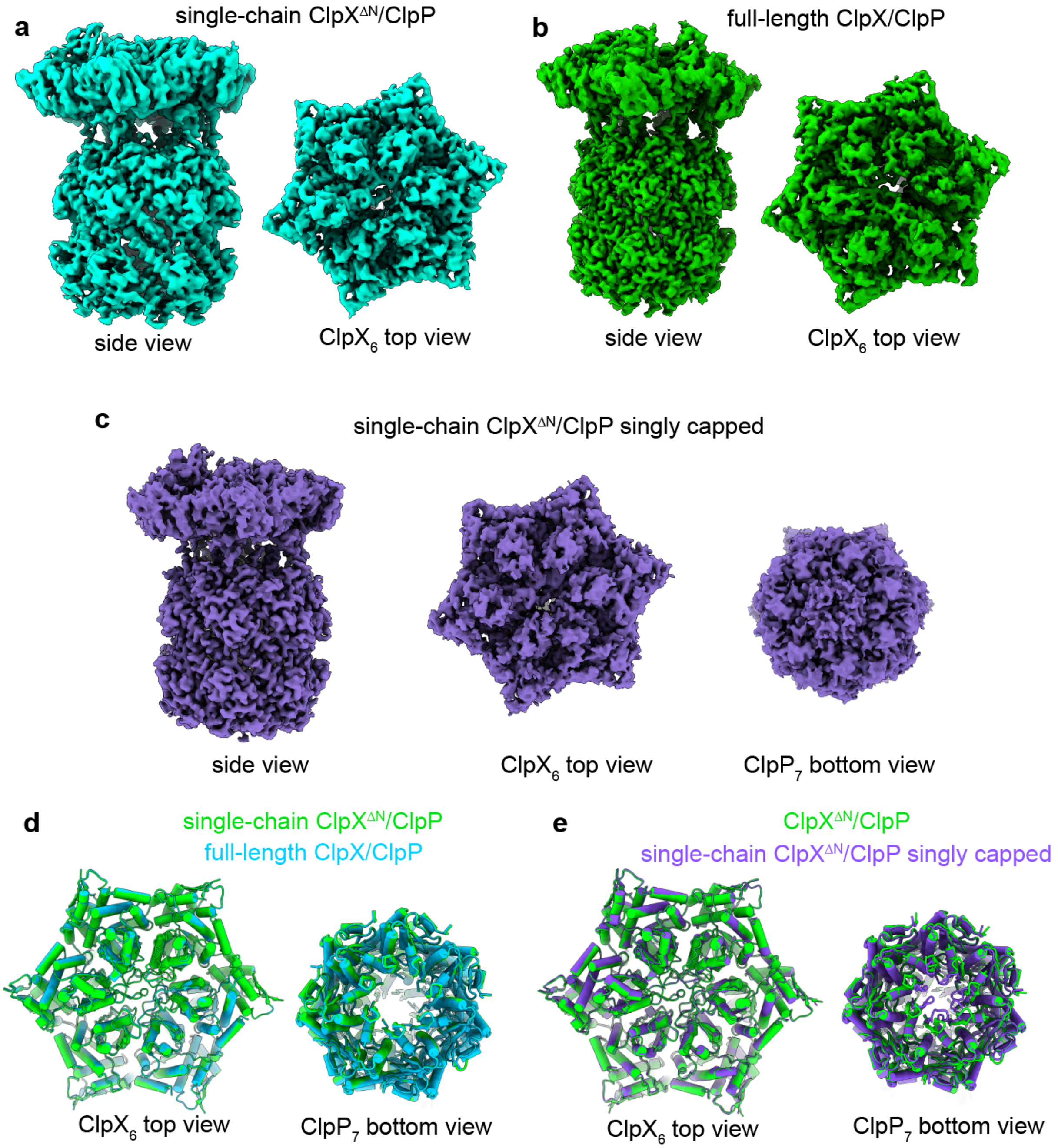

Supplementary Figure 6. Density maps and models. (**a**) Cryo-EM density of single-chain ClpX$^{\Delta N}$/ClpP in side and top views. (**b**) Cryo-EM density of full-length ClpX/ClpP in side and top views. (**c**). Cryo-EM density of singly capped single-chain ClpX$^{\Delta N}$/ClpP in side, top, and bottom views. (**d**). Alignment of full-length ClpX/ClpP (cyan; cartoon representation) and single-chain ClpX$^{\Delta N}$/ClpP atomic models (green; cartoon representation). These structures differ by a Cα RMSD of ~0.3 Å. (**e**) Alignment of ClpX$^{\Delta N}$/ClpP (green; cartoon representation) and exclusively singly capped particles (purple; cartoon representation). These structures differ by a Cα RMSD of ~0.9 Å. Note the axial portal of the distal ClpP ring is closed in the exclusive singly capped ClpX$^{\Delta N}$/ClpP particles (purple model) but open in the predominantly doubly capped ClpX$^{\Delta N}$/ClpP particles (green model).

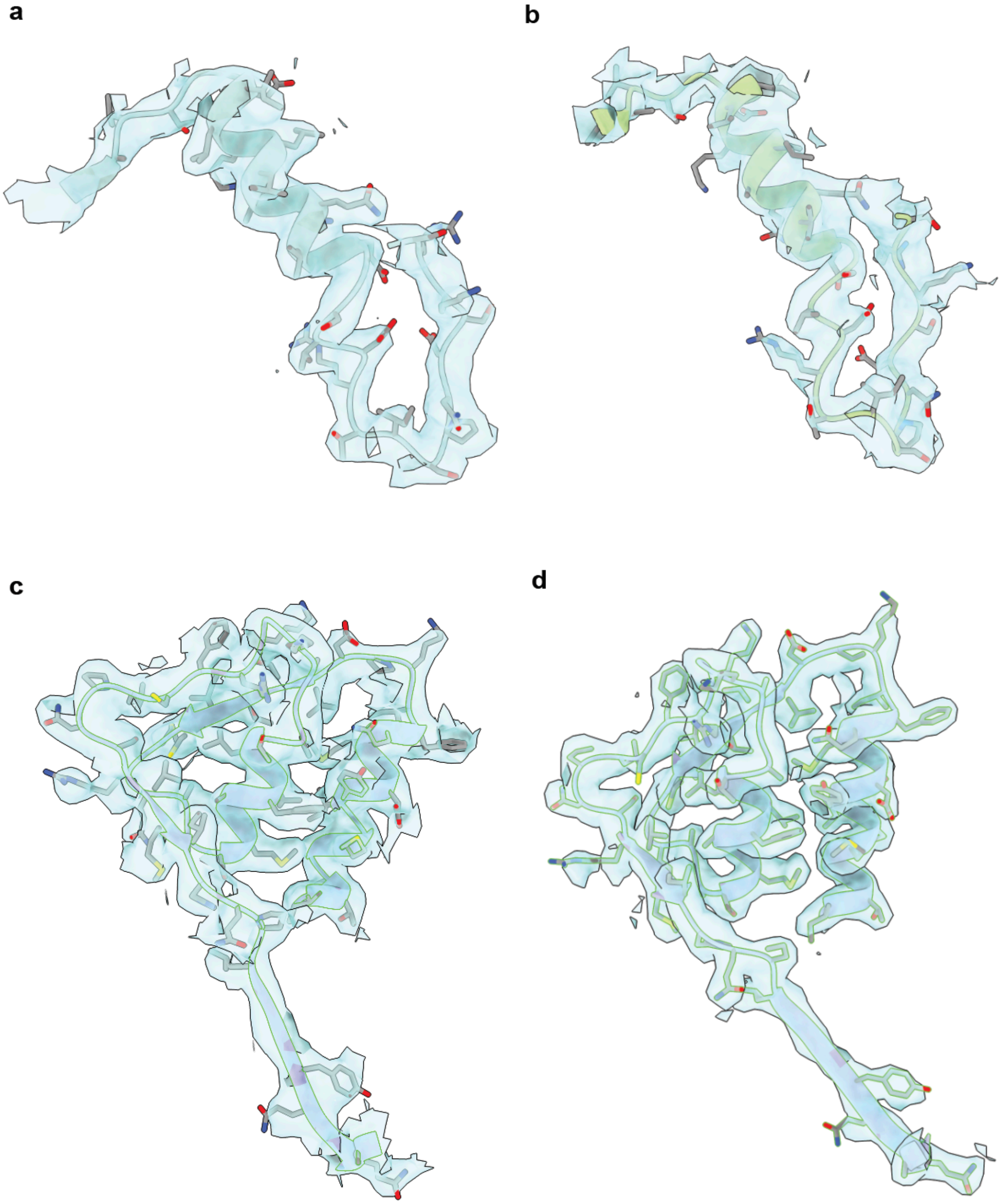

Supplementary Figure 7. Exemplar density maps and fitted atomic models. Cryo-EM density map surrounding ClpX residues 190-220 and fitted atomic models for (**a**) ClpX$^{\Delta N}$/ClpP (pdb 8E8Q) and (**b**) full-length ClpX/ClpP (pdb 8E91). Cryo-EM density map surrounding ClpP residues 70-132 and fitted atomic models for (**c**) ClpX$^{\Delta N}$/ClpP (pdb 8E8Q) and (**d**) full-length ClpX/ClpP (pdb 8E91).

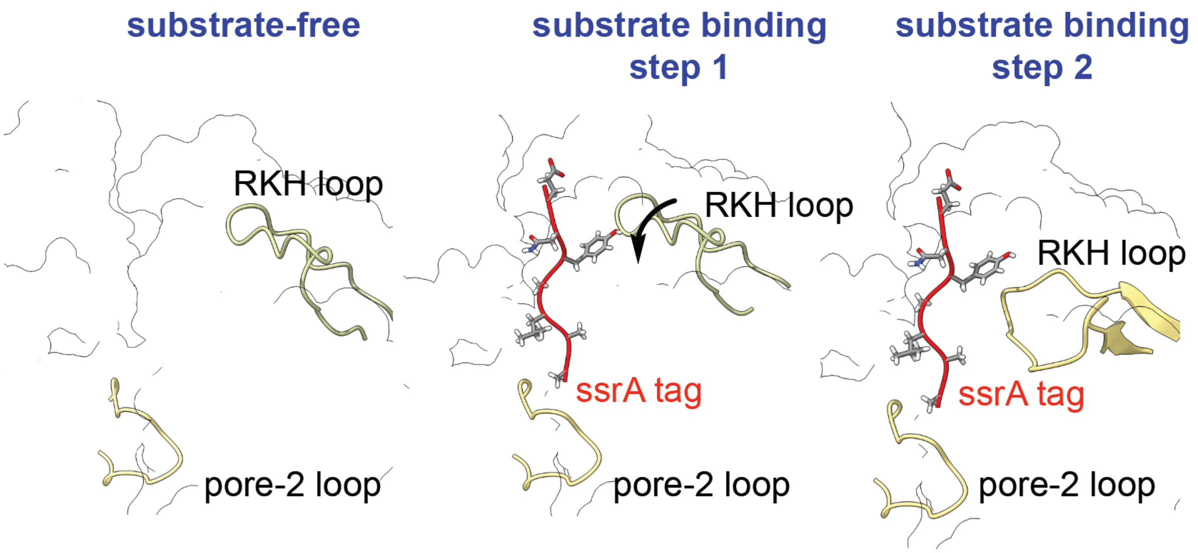

Supplementary Figure 8. Model of two-step ssrA-tag recognition by ClpX. Substrate-free structure of ClpX/ClpP showing the arrangement of RKH and pore-2 loops in stick representation before substrate binding. The ClpX RKH loop (subunit C) had a conformation that would clash with the bound ssrA degron (red, stick representation), suggesting a two-step binding process. Step 1 involves the C-terminus of the ssrA tag binding to the pore-2 loops of ClpX, possibly displacing the RKH loop of chain C. In the second step, downward movement of subunit RKH loop stabilizes the bound conformation of the ssrA degron.

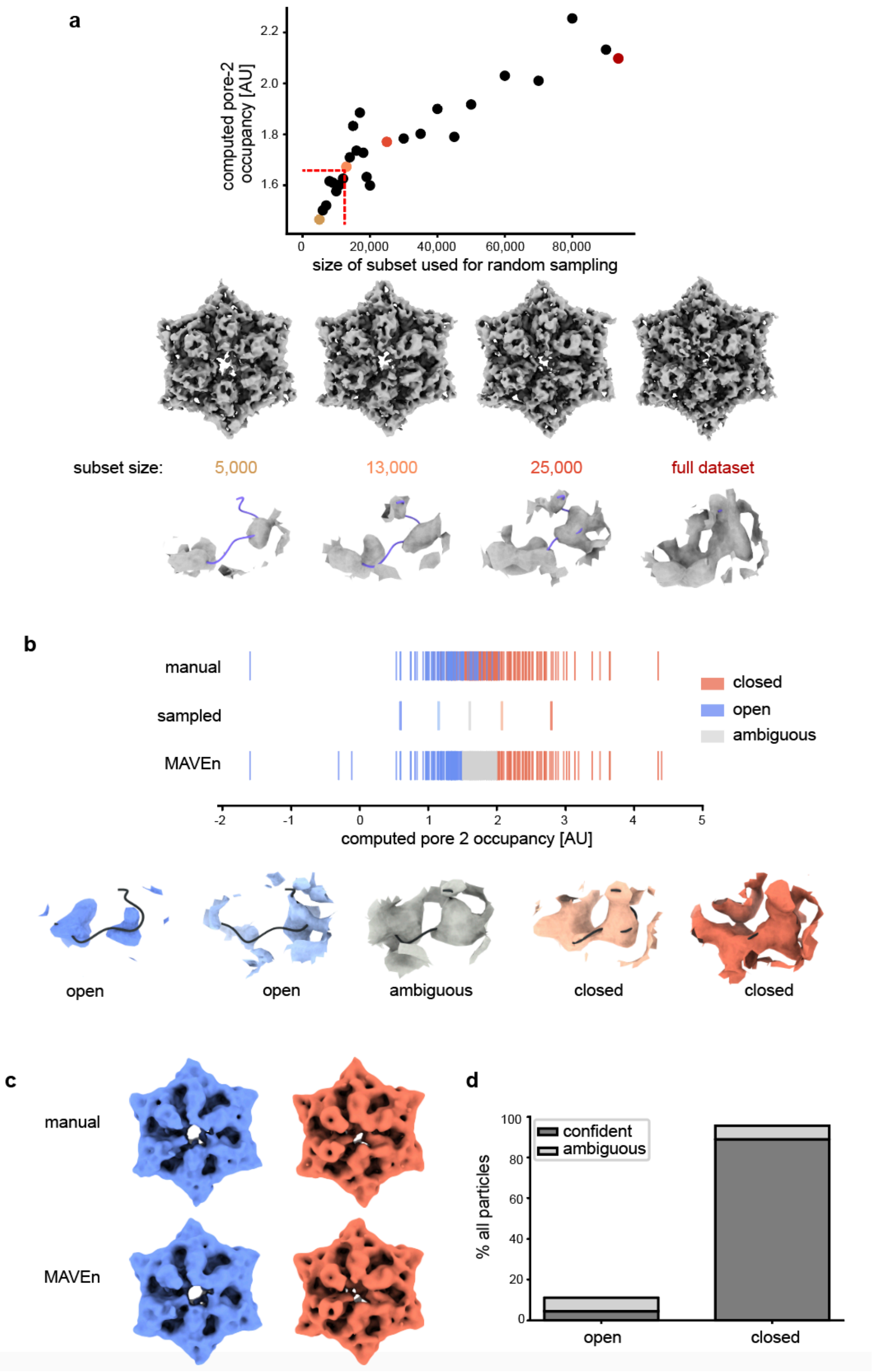

Supplementary Figure 9. Substrate-free ClpX equilibrates between open- and closed-channel conformations. (**a**) After training a cryoDRGN model using particles from the doubly capped ClpXΔN/ClpP data set, occupancy of the pore-2 loop was computed for each particle on a low-resolution map using MAVEn[16,46]. Particles were ranked by this pore-2 occupancy metric and 27 particle subsets were defined using this rank ordering, with each successive subset including additional particles with greater pore-2 loop occupancy. From each of these subsets, 5,000 particles were randomly selected and provided to cryoSPARC for homogeneous refinement. The resulting volumes were queried for pore-2 occupancy at full resolution, revealing a positive correlation between pore-2 occupancy observed in the full resolution maps

and subset ordering, consistent with accurate pore-2 occupancy estimation by MAVEn. Exemplar subsets from the 5,000, 13,000, 25,000, or 93,362 particles with the lowest normalized pore-2 occupancy are colored in shades of orange. Density maps produced by cryoSPARC are depicted for these highlighted subsets (middle). Inset focuses on density for chain A residues 192-199 (bottom). (**b**) As a method to identify open-channel conformations orthogonal to MAVEn, cryoDRGN was used to generate 250 volumes, sampled at k-mean centroid locations, from the 12,500-particle subset, which we expected to contain an approximately 1:1 mixture of open-channel and closed-channel particles. The resulting full resolution maps were classified as open-channel, closed-channel, or ambiguous by MAVEn or manual expert-guided assignment, revealing a strong correspondence between the automated and manual approaches (top). Example volumes sampled across the range of computed pore-2 occupancy values are depicted below and highlight density surrounding chain A residues 192-199. (**c**) CryoSPARC-based homogeneous refinements of 4,139 particles labeled as open by MAVEn or of 4,402 particles labeled as open by manual assignment, alongside homogeneous refinements of size-matched particle stacks sampled randomly from all closed-channel particles. (**d**) Percentage of full particle stack estimated to be open- and closed- pore by MAVEn. Light gray indicates potential range based on particles unclassified by MAVEn. Source data are provided as a Source Data file.

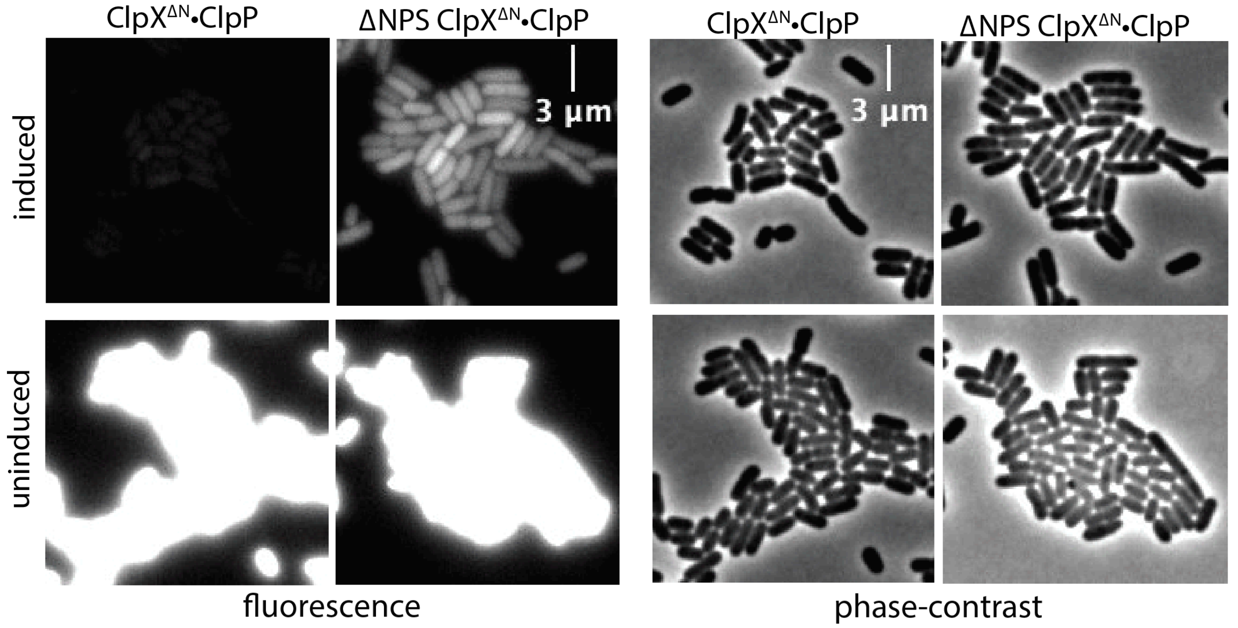

Supplementary Figure 10. Fluorescence (left) and phase-contrast images (right) of *E. coli* T7 Express *ΔclpAΔclpPΔclpX* cells harboring a ProD-GFP-ssrA* plasmid co-transfected with pBAD plasmids encoding ClpX$^{\Delta N}$/ClpP or ΔNPS ClpX$^{\Delta N}$/ClpP plus or minus 80 mM L-arabinose, which induces expression of the ClpX and ClpP variants. Source data are provided as a Source Data file.

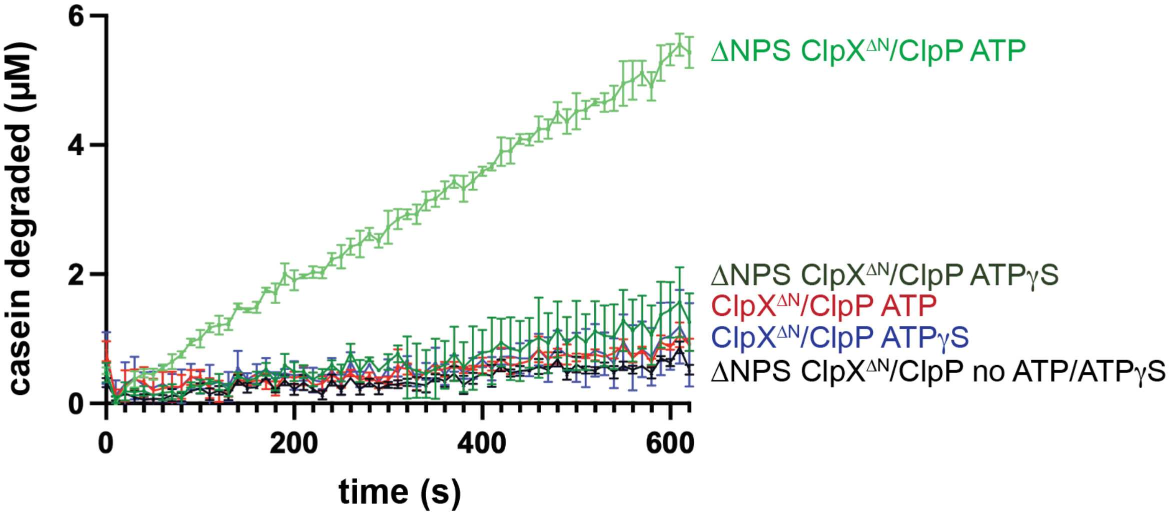

Supplementary Figure 11. The kinetics of FITC-casein (50 μM) degradation by ΔNPS ClpX^{ΔN}/ClpP or ClpX^{ΔN}/ClpP (0.5 μM ClpX variant; 1.5 μM ClpP) were assayed by relief of auto-quenching in the presence of 4 mM ATP, 4 mM ATPγS, or no nucleotide triphosphate at 30 °C. Data points are means (n=3 independent experiments) ± 1 SD. Source data are provided as a Source Data file.

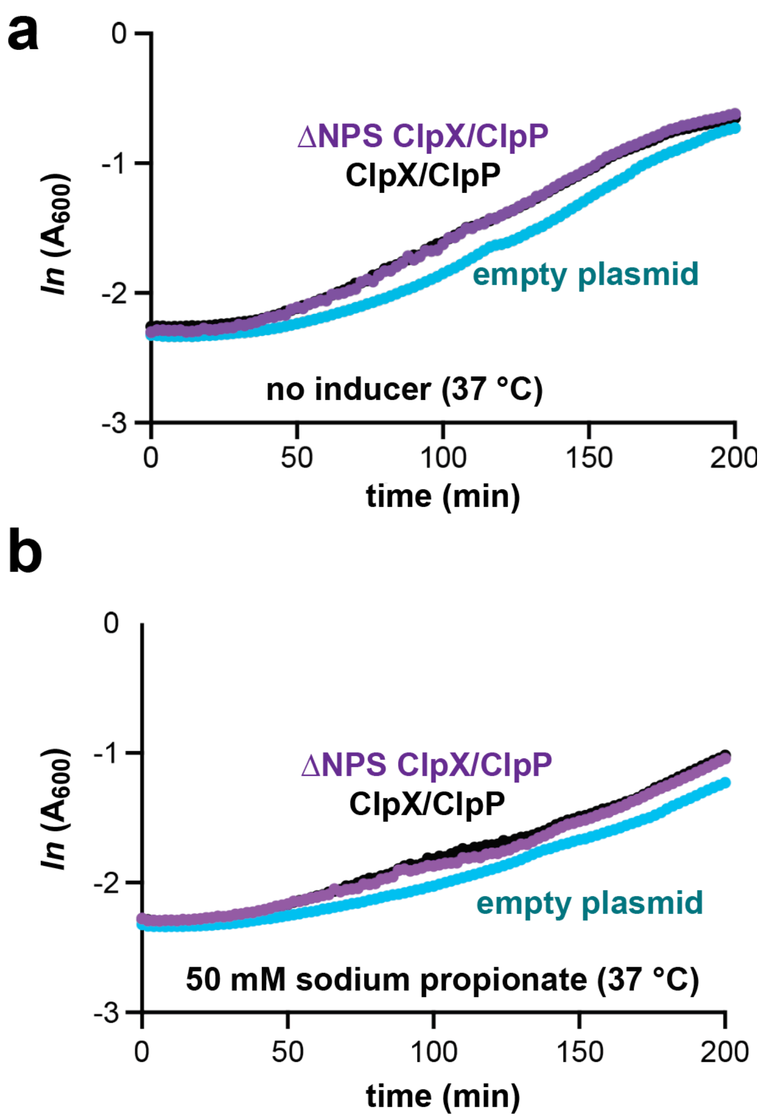

Supplementary Figure 12. Growth curves at 37 ˚C for *E. coli* W3110 expressing wild-type ClpX/ClpP, ∆NPS ClpX/ClpP, or containing an empty vector (pPro33) in the presence of no inducer (**a**) or 50 mM sodium propionate (**b**). Data points are mean values (n=3 technical replicates). Source data are provided as a Source Data file.

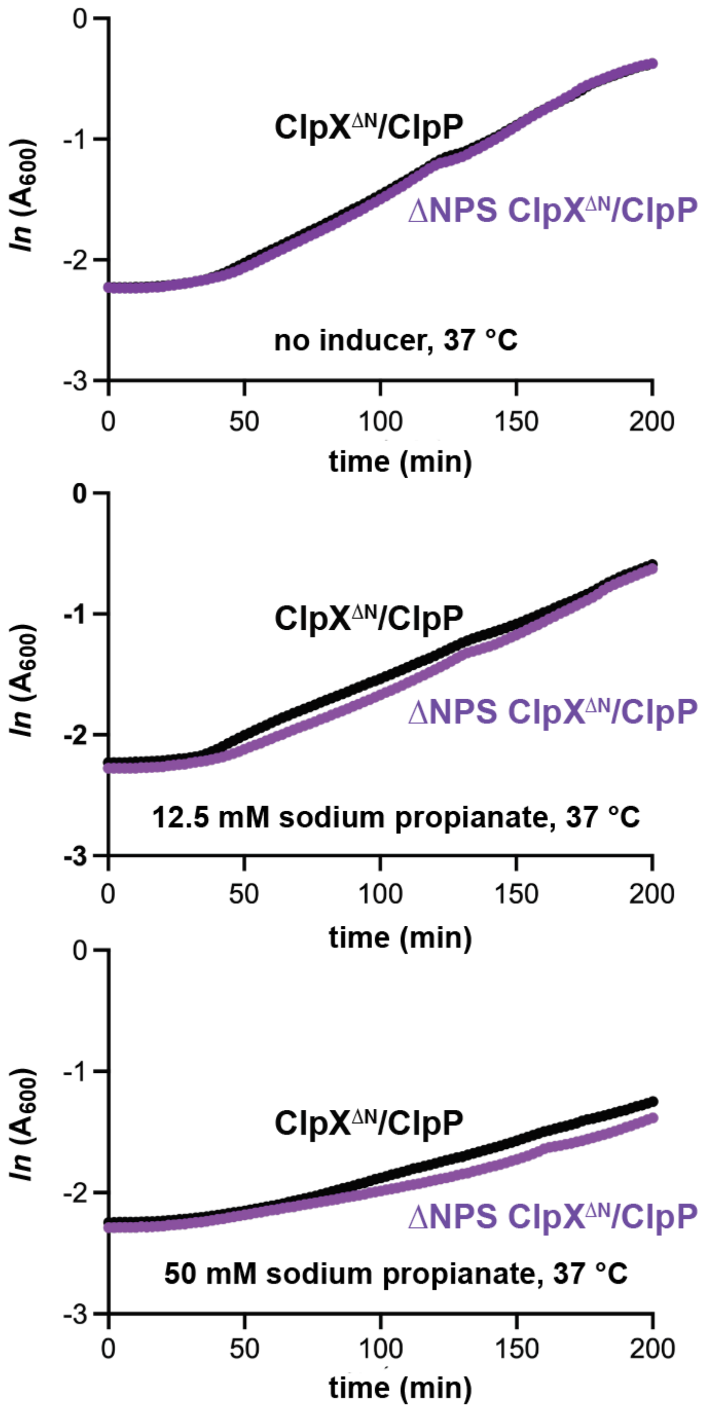

Supplementary Figure 13. Growth curves at 37 ˚C for *E. coli* W3110 expressing $ClpX^{\Delta N}$/ClpP or ΔNPS $ClpX^{\Delta N}$/ClpP in the absence of inducer (top) or presence of sodium propionate inducer at concentrations of 12.5 mM (middle) or 50 mM (bottom). Data points are mean values (n=3 technical replicates). Source data are provided as a Source Data file.

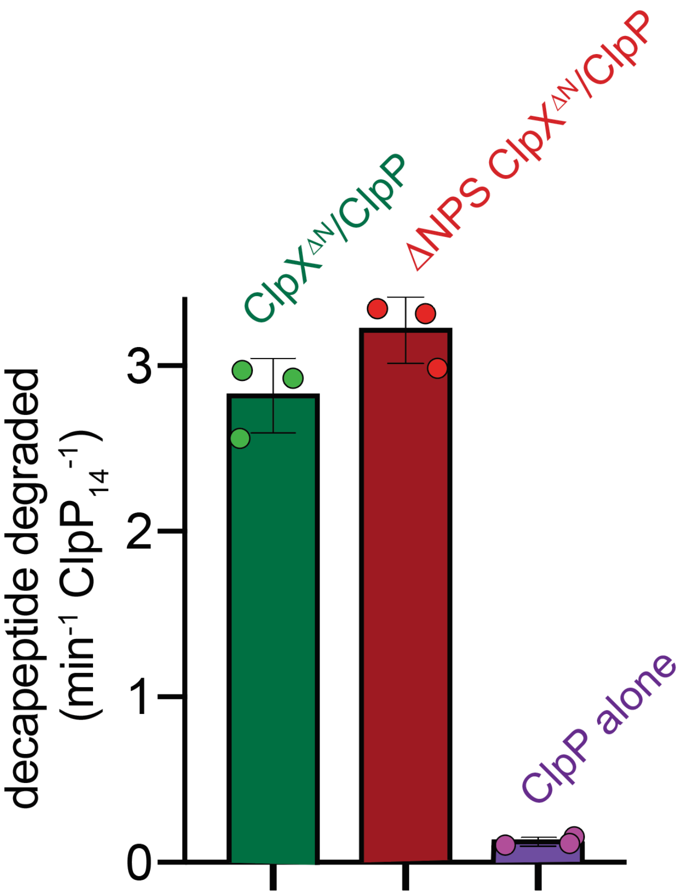

Supplementary Figure 14. Rates of ClpP cleavage of Abz-KASPVSLGY^{NO2}D (15 µM) were assayed by increased fluorescence in the presence/absence of ClpX$^{\Delta N}$ variants (1 µM) and/or ClpP (50 nM). Bars represent averages (n = 3 independent experiments) ± 1 SD with individual data points shown as symbols. Source data are provided as a Source Data file.

Supplementary Figures and Tables

Supplementary Table 1. Cryo-EM data collection, processing, model building, and validation statistics.

Sample and data deposition information			
Sample name	**full-length ClpX/ClpP**	**single-chain ClpX$^{\Delta N}$/ClpP**	**singly capped single-chain ClpX$^{\Delta N}$/ClpP**
Nucleotide added	ATPγS	ATP	ATP
PDB ID	8E91	8E8Q	8E7V
EMDB ID	27952	27946	27941
EMPIAR ID	11706	11707	
Data collection			
Microscope	Titan Krios G3i		
Camera	Gatan K3 direct detection camera (counting mode)		
Magnification (nominal)	130,000 X	105,000 X	
Accelerating voltage (kV)	300		
Total electron dose (e^-/Å^2)	52.7	49.4	
Defocus range (µm)	-0.3 to -1.75	-0.75 to -2.5	
Micrographs collected	11,719	2,405	
Pixel size (Å) initial/calibrated	0.679 / 0.654	0.435 / 0.416	
Map reconstruction			
Image processing package	cryoSPARC		
Total extracted particles	1,899,328	367,632	
Final particle count	214,397	171,869	49,824
Symmetry imposed	C1		
Resolution (Å)			
0.143 GSFSC unmasked	3.1	3.9	4.2
0.143 GSFSC spherical mask	2.8	3.5	3.8
0.143 GSFSC tight/local mask	2.6	3.1	3.1
3DFSC sphericity (out of 1.0)	0.99	0.97	0.96
Model composition			
Non-hydrogen atoms	70,125	70,103	70,085
Protein residues	4,783	4,782	4,779
Ligands	9	9	9
Model refinement			
Refinement package	Phenix		
Map-to-model cross correlation	0.83	0.80	0.82
RMS deviations bond lengths (Å)	0.003	0.003	0.004
RMS deviations bond angles (°)	0.650	0.625	0.665
Model validation			
MolProbity score	0.91	0.87	0.83
Clash score	1.63	1.40	1.18
C-beta outliers (%)	0	0	0
Rotamer outliers (%)	0	0	0
Ramachandran favored (%)	99.2	99.2	99.4
